# Platelet-Rich Plasma Versus Corticosteroid Injections for Chronic Tendinopathies: A Systematic Review and Meta-Analysis

**DOI:** 10.7759/cureus.76051

**Published:** 2024-12-20

**Authors:** Ahmed Elnewishy, Abdelfatah M Elsenosy, Hagar Teama, Naoum Symeon, Ahmed Hamada

**Affiliations:** 1 Trauma and Orthopaedics, Royal Berkshire Hospital, Reading, GBR; 2 Trauma and Orthopaedics, University Hospitals Dorset, Poole, GBR; 3 Pharmacy, Kafr Elsheikh Hospital, Kafr Elsheikh, EGY; 4 Orthopedics and Trauma Surgery, 251 Hellenic Air Force General Hospital, Athens, GRC; 5 Trauma and Orthopaedics, Royal Devon and Exeter University Hospital, Devon, GBR

**Keywords:** ases score, chronic tendinopathy, constant-murley score, corticosteroid injections, dash score, functional recovery, pain reduction, platelet-rich plasma, platelet-rich plasma (prp), visual analog scale

## Abstract

Chronic tendinopathy is a degenerative condition characterized by persistent pain, functional limitations, and tendon thickening, often resulting from repetitive overuse or failed tendon healing. Left untreated, it can progress to tendon rupture, prolonged disability, and impaired quality of life. Management typically includes conservative therapies, such as physical therapy, corticosteroid (CS) injections for short-term relief, and platelet-rich plasma (PRP) as a regenerative therapy with potential long-term benefits. This systematic review and meta-analysis evaluates and compares the effectiveness of PRP and CS injections for chronic tendinopathies regarding pain reduction, functional improvement, and long-term outcomes. A structured search of PubMed, Scopus, Google Scholar, and the Cochrane Library identified randomized controlled trials (RCTs) and comparative studies. Primary outcomes included pain reduction and functional improvement while secondary outcomes assessed heterogeneity and publication bias. PRP and CS injections demonstrated no significant difference in short-term pain reduction. Functional outcomes were comparable in the short term but showed a trend favoring PRP at longer follow-up periods. PRP exhibited a slight advantage in long-term functional recovery, though the results were not statistically significant. Substantial heterogeneity across studies highlights the need for standardized protocols and larger trials to confirm these findings and optimize treatment strategies.

## Introduction and background

Introduction

Chronic tendinopathies are degenerative conditions of tendons, characterized by pain, swelling, and functional impairment. They result from complicated interactions among mechanical, biochemical, and systemic factors that result in failed tendon recuperation and structural degeneration [1]. The prevalence and incidence of chronic tendinopathies were stated as 16.6 and 7.9 per 1,000 registered patients, respectively. This information highlights the sizeable burden of tendinopathies [2].

Reasons for chronic tendinopathies include both intrinsic and extrinsic factors. Intrinsic elements, including getting old, genetic predispositions, and systemic situations like diabetes, contribute to reduced tendon resilience and healing ability [3]. Diabetes is specifically associated with multiplied risk due to continual irritation and increased glucose degrees that impair tendon mobility features and recovery [4]. Extrinsic chance factors encompass repetitive mechanical overload, wrong training strategies, and overuse throughout sports or occupational activities. These external stresses result in microtrauma and exacerbate degenerative modifications in tendons [5].

The pathophysiology of chronic tendinopathies is multifaceted and entails a complicated interplay among mechanical overload, cell adjustments, and biochemical approaches. This involves multiplied expression of vascular endothelial growth factor (VEGF) and extraordinary angiogenic mediators, contributing to hypervascularization and neighborhood nerve sensitization. These modifications are related to tendon pain and impaired features [6]. An important issue of tendinopathy is the dysregulation of the tendon extracellular matrix (ECM). This dysregulation results in improved production of matrix metalloproteinases (MMPs) and decreased degrees of tissue inhibitors of metalloproteinases (TIMPs), leading mainly to collagen breakdown and impaired ECM integrity [7]. Additionally, tendinopathic tendons show accelerated cellularity, with tenocytes adopting a rounded phenotype, indicative of impaired features and failed transformation [8].

Moreover, oxidative pressure plays a pivotal role in tendinopathy pathogenesis. Mechanical overload generates reactive oxygen species (ROS), which harm tendon cells and ECM components. In vitro research has shown that oxidative stress impairs tendon recovery by inducing cell apoptosis and inhibiting collagen synthesis [9]. Chronic low-grade inflammation additionally contributes to the pathophysiology of tendinopathy. While acute irritation is absent, accelerated degrees of pro-inflammatory cytokines, such as interleukin-1β (IL-1β) and tumor necrosis factor-alpha (TNF-α), have been determined in tendinopathic tissues. These cytokines disrupt the stability of tendon anabolism and catabolism, promoting tissue degeneration [3].

Non-invasive remedies continue to be the primary-line approach for dealing with persistent tendinopathies, with a focus on assuaging signs and symptoms, improving features, and promoting tendon healing. Among these, eccentric exercise therapy has confirmed the most steady effectiveness throughout multiple research. This approach emphasizes controlled lengthening of the muscle-tendon unit, stimulating collagen synthesis, and improving tendon shape and function [10]. Extracorporeal surprise wave remedy (ESWT) is another extensively followed non-invasive remedy. ESWT promises mechanical electricity to the affected tendon, promoting angiogenesis, lowering pain, and stimulating collagen production. It has proven massive efficacy in conditions like Achilles tendinopathy and plantar fasciitis, supplying a viable alternative while conventional methods fail [11].

Additionally, therapeutic ultrasound and low-level laser remedies have shown promise in improving pain and other characteristics. These modalities enhance cell restoration and modulate irritation, though their effectiveness varies based on the affected person and type of tendon [12]. Dry needling, which involves growing microtrauma to stimulate healing, has emerged as a minimally invasive opportunity for tendinopathy. Research suggests its use for improving signs and promoting localized tendon restoration, although more elaborate comparative studies are sought [13].

Nonsteroidal anti-inflammatory drugs (NSAIDs) are generally prescribed for handling pain and infection associated with tendinopathies. While tendinopathies are now identified as predominantly degenerative instead of inflammatory conditions, NSAIDs continue to be effective for quick-time period pain alleviation, especially throughout acute exacerbations. Both oral and topical formulations can be used, with topical NSAIDs imparting targeted alleviation with fewer systemic side effects [12]. However, the long-term use of NSAIDs is discouraged due to adverse consequences, inclusive of gastrointestinal and cardiovascular dangers. Additionally, there is little evidence suggesting that NSAIDs may impair tendon recuperation by disrupting the inflammatory technique necessary for tissue restoration [14].

Surgical intervention is normally reserved for extreme or refractory cases of persistent tendinopathy where non-invasive remedies fail. The primary goal of surgical treatment is to do away with degenerative tissue, stimulate restoration, and restore tendon features. Techniques vary based on the affected tendon; however, they typically include open debridement, arthroscopic tactics, or minimally invasive tactics such as percutaneous tenotomy. For instance, in non-insertional Achilles tendinopathy, minimally invasive debridement or plantaris tendon release is increasingly preferred due to reduced recovery time and decreased costs [15]. In cases wherein tendon rupture is imminent or has already occurred, surgical repair or tendon transfer can be required. The achievement of surgical options depends on early analysis, suitable patient choice, and adherence to post-surgical rehabilitation protocols [16].

Platelet-rich plasma (PRP) is a focused type of plasma derived from autologous blood, characterized by an excessive concentration of platelets. Platelets in PRP contain numerous growth factors, which include platelet-derived growth factor (PDGF), transforming growth factor-beta (TGF-β), and vascular endothelial growth factor (VEGF), which play a key role in promoting tissue restoration and regeneration [17]. PRP is received by centrifuging whole blood to isolate and concentrate the platelet-wealthy fraction, which is then activated to release bioactive molecules [18]. The therapeutic intent for PRP lies in its capability to supply supra-physiological concentrations of growth factors and cytokines to the site of injury, enhancing the frame’s natural recovery strategies [19].

PRP’s therapeutic outcomes are frequently attributed to its growth factor content, which modulates inflammation, enhances cell proliferation, and promotes angiogenesis. This makes it particularly effective in treating musculoskeletal injuries, including tendinopathies, cartilage damage, and ligament sprains [20]. In chronic tendinopathies, PRP has shown potential in promoting collagen synthesis and restoring tendon structure, providing a regenerative stimulus to damaged tissues [21]. However, mild adverse effects, such as temporary pain, swelling, or local irritation at the injection site, can occur; more serious complications remain rare.

Studies also display that PRP reduces pain and improves features in conditions consisting of lateral epicondylitis and patellar tendinopathy by regulating pro-inflammatory cytokines and promoting the restoration of micro-tears [22]. Moreover, PRP enhances the vascularization of damaged tissues, which is crucial for delivering nutrients and oxygen to facilitate recovery [23]. Despite its substantial use, the therapeutic efficacy of PRP can vary due to differences in guidance protocols, platelet attention, and activation techniques. Standardization of those factors is essential to maximizing its clinical blessings [24].

Corticosteroids are a category of steroid hormones that are synthesized by way of the adrenal cortex or created artificially for therapeutic use. They encompass glucocorticoids, which affect metabolism and immune responses, and mineralocorticoids, which alter electrolyte and water balance. Synthetic corticosteroids, inclusive of prednisone and dexamethasone, mimic the outcomes of endogenous hormones and are extensively utilized in treating inflammatory, allergic, and autoimmune diseases [25]. These medications exert their outcomes by modulating gene transcription via binding to glucocorticoid receptors, influencing several physiological methods, together with immune homeostasis and inflammation regulation [26].

Corticosteroids are widely diagnosed for their strong anti-inflammatory and immunosuppressive properties. They decrease the manufacture of inflammatory mediators consisting of prostaglandins and cytokines while suppressing immune cell activity. These consequences make corticosteroids powerful for conditions starting from asthma and rheumatoid arthritis to inflammatory bowel disease and chronic tendinopathies [27]. In tendinopathies, corticosteroid injections are regularly used to alleviate pain and decrease swelling, offering speedy comfort with the aid of inhibiting the inflammatory cascade [28].

Corticosteroids were extensively used in the remedy of persistent tendinopathies, in most cases for their anti-inflammatory and pain-relieving consequences. These pills suppress the production of pro-inflammatory cytokines and other inflammatory mediators, thereby reducing pain and swelling in affected tendons [29]. Corticosteroid injections are especially powerful for brief-time period pain management in conditions such as rotator cuff tendinopathy, lateral epicondylitis, and Achilles tendinopathy. Studies have shown a considerable decrease in pain and improved range of movement within weeks of corticosteroid management [30]. Despite their advantages, corticosteroids have restricted lengthy-time period effectiveness and potential negative consequences. Repeated use in tendinopathies has been linked to deleterious results on tendon shape, consisting of weakened collagen integrity and increased danger of rupture [31]. The effectiveness of PRP versus corticosteroid injections for persistent tendinopathies remains doubtful, with inconsistent consequences suggested across research. This highlights the want for a scientific analysis to clarify their relative blessings.

Review objective

This review aimed to compare the use of PRP and corticosteroid injections for managing chronic tendinopathies, focusing on pain relief and functional improvement to guide evidence-based treatment.

## Review

Methods

Search Strategy

A structured search was conducted in November 2024 using PubMed, Scopus, Google Scholar, and the Cochrane Library to identify studies comparing PRP and corticosteroid injections for chronic tendinopathies. The search utilized a combination of MeSH terms and keywords, including “platelet-rich plasma”, “PRP”, “corticosteroid injections”, “tendinopathy”, “rotator cuff”, and “shoulder pain”. Boolean operators (AND, OR) were applied to refine the results, and filters were set to restrict the search to English-language studies. Additionally, reference lists of the identified articles were reviewed to capture any relevant studies not retrieved during the initial search.

Inclusion Criteria

Studies were included if they were randomized controlled trials (RCTs) or prospective comparative studies directly comparing PRP and corticosteroid injections for chronic tendinopathies. Eligible studies had to report at least one primary outcome, such as pain reduction (measured by the visual analog scale (VAS)) or functional improvement (measured by tools such as Disabilities of the Arm, Shoulder, and Hand (DASH), American Shoulder and Elbow Surgeons (ASES), or Constant-Murley scores). Only studies published in English and conducted on adult human populations were included.

Exclusion Criteria

Studies were excluded if they did not directly compare PRP and corticosteroid injections. Case reports, case studies, editorials, opinion pieces, and conference abstracts were not considered. Additionally, studies were excluded if they did not report sufficient outcome data or were published in a language other than English.

Outcome Measures

The primary outcomes evaluated in the analysis included pain reduction, measured by the VAS, and functional improvement, assessed using standardized tools such as the DASH score, Constant-Murley score, and ASES score. Secondary outcomes included range of motion (ROM), imaging-confirmed tendon healing, and patient-reported satisfaction.

Data Extraction and Quality Assessment

Data were extracted independently by two reviewers using a standardized form to record information on study design, patient demographics, interventions, outcomes, and follow-up durations. Disagreements were resolved by consensus or by consulting a third reviewer. The quality of included studies was assessed using the Cochrane Risk of Bias Tool 2.0 (RoB 2), focusing on domains such as randomization, deviations from intended interventions, missing outcome data, outcome measurement, and selection of reported results. Studies were categorized based on their overall risk of bias as low risk, some concerns, or high risk.

Statistical Analysis

Statistical analysis was conducted using RevMan version 5.4 software (Cochrane Collaboration, London, UK) [32]. For continuous outcomes, such as pain reduction (VAS scores) and functional improvement (ASES, Constant-Murley, and DASH scores), standardized mean differences (SMDs) with 95% confidence intervals (CIs) were calculated. Heterogeneity among studies was assessed using the I² statistic, with values above 50% indicating substantial heterogeneity. A fixed-effects model was used if heterogeneity was low while a random-effects model was applied when heterogeneity was significant. Funnel plots and Egger’s test were utilized to evaluate publication bias, with statistical significance set at p < 0.05.

Results

Search and Study Selection

A structured search was performed to pick out research evaluating PRP injections with corticosteroid injections for rotator cuff tendinopathy. The initial search yielded 150 records, with 20 duplicates eliminated, leaving 130 records for screening. During the title and abstract review, 85 studies were excluded, as they did now not meet the inclusion criteria, which targeted studies comparing PRP and corticosteroid remedies for rotator cuff tendinopathy, partial tears, or shoulder impingement syndrome.

**Figure 1:**
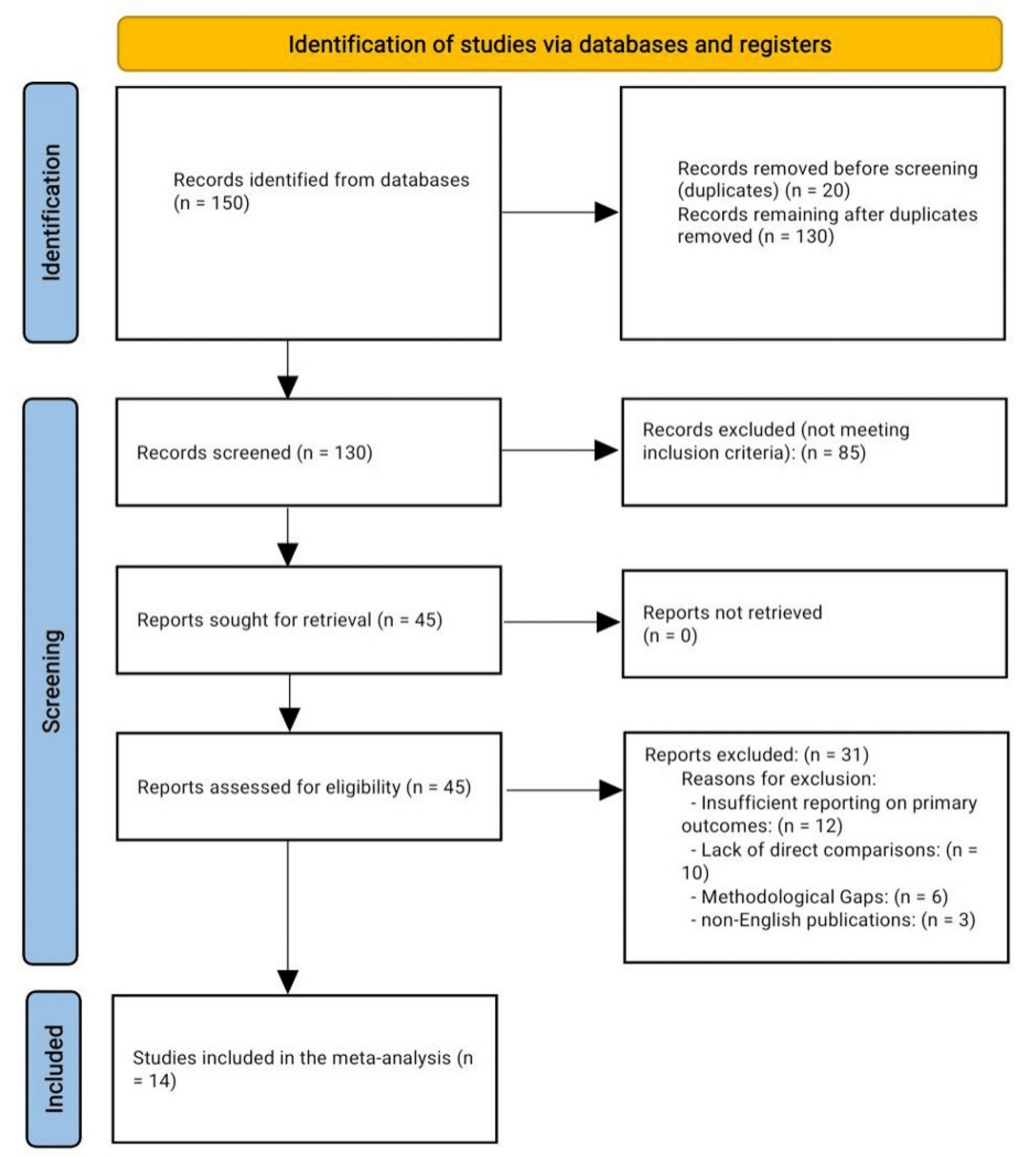
PRISMA flow chart for the included studies PRISMA: Preferred Reporting Items for Systematic Reviews and Meta-Analyses

Following this, 45 full-text articles were assessed for eligibility. After an in-depth assessment, 31 studies were excluded due to motives, which include insufficient reporting of primary effects, methodological barriers, and non-English publications. Ultimately, 14 studies fulfilled all inclusion criteria and were included in the final synthesis. A detailed flow diagram of the study selection is illustrated in Figure 1.

Study Characteristics

This systematic review and meta-analysis encompasses 14 randomized controlled trials comparing PRP injections with corticosteroid interventions. The cumulative sample size comprised 918 individuals, with an individual look at populations ranging from 30 to 120 subjects (mean: 65.6 ± 24.3). The methodological quality was predominantly excessive, with eight studies categorized as Level I evidence, 5 research as Level II evidence, and 1 study as Level III evidence, in step with the Oxford Centre for Evidence-Based Medicine standards. The demographic composition verified a heterogeneous population with mean age ranges of 41.85 ± 10.21 to 57.33 ± 9.80 years. Gender distribution exhibited substantial variation throughout studies, with female representation ranging from 19% to 81%. The primary pathologies investigated included rotator cuff tendinopathy, partial tears, subacromial impingement syndrome, and calcific tendinitis, with maximum studies that specialize in persistent presentations.

The interventions consisted of standardized PRP arrangements (2-6 mL) administered through diverse protocols (single as opposed to double centrifugation) and corticosteroid injections (predominantly methylprednisolone, triamcinolone, or betamethasone mixed with local anesthetic). Ultrasound-guided administration was carried out in the majority of studies, ensuring particular healing delivery. Follow-up periods established extensive heterogeneity, ranging from 2 months to 24 months (median: 6 months), with systematic evaluation periods at 1, 3, and 6 months submit-intervention. Outcome measures integrated tested assessment gear, along with pain quantification (VAS), functional assessment (DASH, ASES, Constant-Murley Score), and quality of life indices (Western Ontario Rotator Cuff (WORC), 36-Item Short Form Health Survey (SF-36)). Several studies used goal imaging parameters through MRI or ultrasonography to assess structural changes. Adverse occasions were systematically documented, with one taking a look at reporting a higher incidence of adhesive capsulitis inside the PRP cohort (12%) compared to corticosteroid administration (2.6%). This complete characterization of observed parameters enables a strong comparative analysis of therapeutic efficacy between PRP and corticosteroid interventions (Table 1 and Table 2).

**Table 1:** Study design, sample size, and level of evidence PRP: platelet-rich plasma; RC: rotator cuff; SAIS: subacromial impingement syndrome; NACD: needle aspiration of calcific deposits

Study	Study Design	Sample Size	Level of Evidence
Dadgostar et al. [30]	Randomized double-blind clinical trial comparing PRP injections to corticosteroids for RC tendinopathy.	58 (30 PRP, 28 corticosteroid)	Level II
Kwong et al. [32]	Double-blind randomized controlled trial comparing PRP injections with corticosteroid injections for PTRCTs or tendinopathy.	99 (47 PRP, 52 corticosteroid)	Level I
Ibrahim et al. [33]	Randomized controlled trial comparing ultrasound-guided PRP versus corticosteroid injection for RCT	30 (15 PRP, 15 corticosteroid)	Level I
Saleem et al. [34]	Double-blind randomized controlled trial comparing ultrasound-guided PRP and corticosteroid injections for RC tendinopathy	60 (30 PRP, 30 corticosteroid)	Level I
Shams et al. [35]	Prospective randomized controlled trial comparing subacromial PRP injections to corticosteroid injections for partial RC tears	40 (20 PRP, 20 corticosteroid)	Level II
Kumar et al. [36]	Prospective randomized comparative study comparing PRP to corticosteroid injections for de Quervain’s tenosynovitis	60 (30 PRP, 30 corticosteroid)	Level II
Say et al. [37]	Prospective comparative study evaluating single-dose PRP vs. steroid injections for SIS	60 (30 PRP, 30 corticosteroid)	Level III
Von Wehren et al. [38]	Prospective comparative study comparing subacromial injections of ACP and cortisone for symptomatic partial RC tears	50 (25 ACP, 25 cortisone)	Level III
Barreto et al. [39]	Double-blind randomized clinical trial comparing subacromial PRP and corticosteroid injections for RC impingement syndrome	51 (25 PRP, 26 corticosteroid)	Level I
Pasin et al. [40]	Randomized comparative study comparing PRP injection, corticosteroid injection, and physical therapy for SAIS	90 (30 PRP, 30 corticosteroid, 30 PT)	Level II
Jo et al. [41]	Randomized controlled trial comparing allogeneic PRP injection with corticosteroid injection for RC disease	60 (30 PRP, 30 corticosteroid)	Level I
Sari & Eroglu [42]	Randomized controlled trial comparing PRP, prolotherapy, corticosteroid, and lidocaine injections for RC lesions	120 (30 per group)	Level I
Oudelaar et al. [43]	Double-blind randomized controlled trial comparing NACD with PRP or corticosteroids for RC calcific tendinitis	80 (41 PRP, 39 corticosteroid)	Level I
Sabaah & Nassif [44]	Randomized controlled trial comparing prolotherapy, PRP, and corticosteroid injections for RC tendinopathy	60 (20 per group)	Level I

**Table 2:** Patient demographics, intervention, and follow-up PRP: platelet-rich plasma; CS: corticosteroid; ACP: autologous conditioned plasma

Study	Mean Age PRP	Mean Age Corticosteroid	Gender PRP (M/F)	Gender Corticosteroid (M/F)	Intervention	Follow-Up Duration
Dadgostar et al. [30]	57.33 ± 9.80	53.60 ± 7.24	19%/81%	81%/19%	PRP: 3cc intra-articular + 3cc tendon tear site. CS: Depo-medrol 40mg + lidocaine.	3 months
Kwong et al. [32]	49.94 ± 9.70	49.08 ± 9.54	31/16	33/19	PRP: Leukocyte-poor PRP (3–5 mL). CS: Triamcinolone + bupivacaine.	12 months
Ibrahim et al. [33]	46.8 ± 10.6	41.5 ± 12.5	Comparable	Comparable	PRP: 2 mL PRP via double-centrifugation. CS: Methylprednisolone acetate + lidocaine.	2 months
Saleem et al. [34]	55.2 ± 5.2	55.9 ± 4.3	6/24	8/22	PRP: 3cc intra-articular + 3cc tendon tear site. CS: Depo-Medrol 40mg + lidocaine.	3 months
Shams et al. [35]	52 ± 12	50 ± 10	10/10	11/9	PRP: MyCells PRP system, 2.5 mL PRP. CS: Triamcinolone acetonide 40 mg.	6 months
Kumar et al. [36]	35.83 ± 8.48	37.80 ± 6.44	73.3%/26.7%	66.7%/33.3%	PRP: Single 3 mL injection. CS: Methylprednisolone acetate 40 mg.	12 months
Say et al. [37]	49.2 ± 7	50.2 ± 2.7	10/20	12/18	PRP: 2.5 mL PRP. CS: Methylprednisolone 40 mg + prilocaine.	6 months
Von Wehren et al. [38]	53 ± 14	55 ± 10	12/13	14/11	PRP: ACP processed using Arthrex system. Cortisone: Triamcinolone 40 mg.	6 months
Barreto et al. [39]	53.2 ± 9.4	53.0 ± 11	42%/58%	28%/72%	PRP: Double centrifugation, 3 mL PRP. CS: Betamethasone dipropionate.	3 months
Pasin et al. [40]	49.4 ± 9.1	47.73 ± 9.6	37% male	37% male	PRP: 4 mL PRP. CS: Triamcinolone 40 mg + lidocaine.	8 weeks
Jo et al. [41]	55.3 ± 10.3	52.5 ± 11.2	36.7% male	30% male	PRP: 4 mL allogeneic PRP. CS: Triamcinolone 40 mg + lidocaine.	6 months
Sari & Eroglu [42]	52.1 ± 10.8	53 ± 9.6	36.7% male	35% male	PRP: Double centrifugation, 5 mL PRP. CS: 2 mL triamcinolone + lidocaine.	24 weeks
Oudelaar et al. [43]	48.8 ± 5.8	48.5 ± 6.3	39% female	41% female	PRP: 6 mL leukocyte-rich PRP. CS: Bupivacaine + triamcinolone.	2 years
Sabaah & Nassif [44]	41.85 ± 10.21	41.85 ± 10.21	70% female	70% female	PRP: 5 mL PRP. CS: Betamethasone + lidocaine.	3 months

Quality Assessment of Included Studies

**Figure 2:**
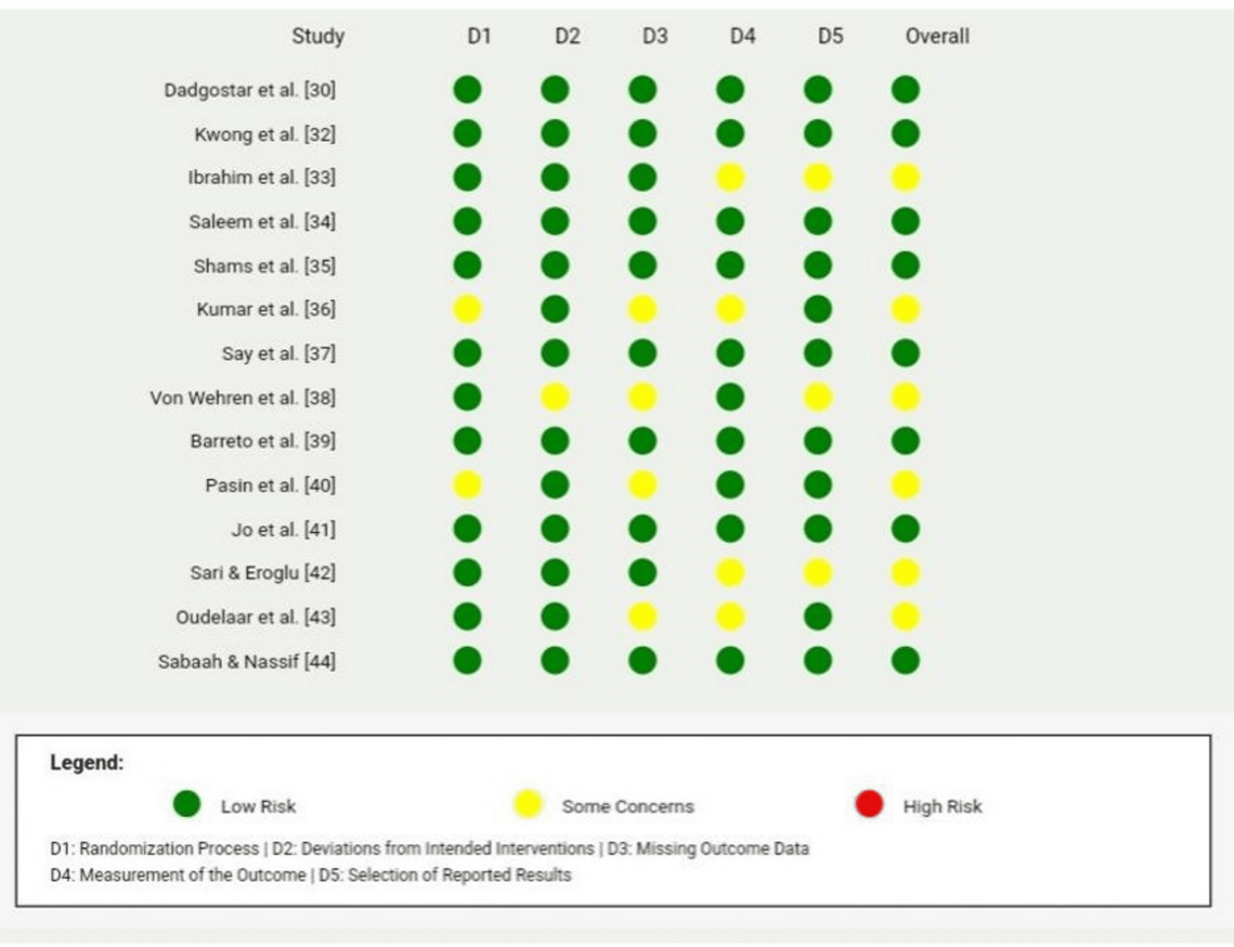
Risk of bias assessment across included studies using the Cochrane RoB2 tool

The included studies were assessed using the Cochrane Risk of Bias Tool 2.0 (RoB2; The Cochrane Collaboration, London, UK), focusing on domains such as randomization, deviations from intended interventions, missing outcome data, outcome measurement, and selection of reported results. Studies were categorized based on their overall risk of bias as low risk, some concerns, or high risk. The assessment revealed variability in quality, with most studies demonstrating a low risk of bias across key domains, although several studies exhibited some concerns in areas such as missing outcome data and outcome measurement. A detailed summary of the quality assessment is presented in Table 3 and Figure 2.

**Table 3:** Quality assessment for included studies using the Cochrane Risk of Bias Tool 2.0 (RoB2)

Study	Randomization Process	Deviations from Intended Interventions	Missing Outcome Data	Outcome Measurement	Selection of Reported Results	Overall Risk of Bias
Dadgostar et al. [30]	Low Risk	Low Risk	Low Risk	Low Risk	Low Risk	Low Risk
Kwong et al. [32]	Low Risk	Low Risk	Low Risk	Low Risk	Low Risk	Low Risk
Ibrahim et al. [33]	Low Risk	Low Risk	Low Risk	Some Concerns	Some Concerns	Some Concerns
Saleem et al. [34]	Low Risk	Low Risk	Low Risk	Low Risk	Low Risk	Low Risk
Shams et al. [35]	Low Risk	Low Risk	Low Risk	Low Risk	Low Risk	Low Risk
Kumar et al. [36]	Some Concerns	Low Risk	Some Concerns	Some Concerns	Low Risk	Some Concerns
Say et al. [37]	Low Risk	Low Risk	Low Risk	Low Risk	Low Risk	Low Risk
Von Wehren et al. [38]	Low Risk	Some Concerns	Some Concerns	Low Risk	Some Concerns	Some Concerns
Barreto et al. [39]	Low Risk	Low Risk	Low Risk	Low Risk	Low Risk	Low Risk
Pasin et al. [40]	Some Concerns	Low Risk	Some Concerns	Low Risk	Low Risk	Some Concerns
Jo et al. [41]	Low Risk	Low Risk	Low Risk	Low Risk	Low Risk	Low Risk
Sari & Eroglu [42]	Low Risk	Low Risk	Low Risk	Some Concerns	Some Concerns	Some Concerns
Oudelaar et al. [43]	Low Risk	Low Risk	Some Concerns	Some Concerns	Low Risk	Some Concerns
Sabaah & Nassif [44]	Low Risk	Low Risk	Low Risk	Low Risk	Low Risk	Low Risk

Results of the meta-analysis

Comparison of PRP and Corticosteroid Injections for Pain Reduction (VAS Score)

**Figure 3:**
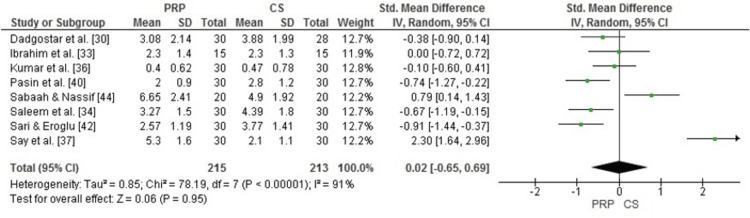
Forest plot comparing PRP and corticosteroid injections for pain reduction (VAS) PRP: platelet-rich plasma; VAS: visual analog scale Source: [30,33,34,36,37,40,42,44]

The forest plot evaluated the effectiveness of PRP compared to corticosteroid (CS) injections in reducing pain, as measured by the VAS. The pooled standard mean difference (SMD) was 0.02 (95% CI: -0.65 to 0.69), which indicated no statistically significant difference between the PRP and CS groups (p = 0.95). However, substantial heterogeneity was observed (I² = 91%), likely due to variations in study design, PRP preparation protocols, and follow-up durations. Individual studies, such as those by Say et al. and Sabaah & Nassif, reported greater improvements with PRP [37,44]. However, the overall pooled analysis suggested comparable efficacy between PRP and corticosteroids for pain reduction (Figure 3).

Publication Bias Assessment for Pain Reduction (VAS Score)

**Figure 4:**
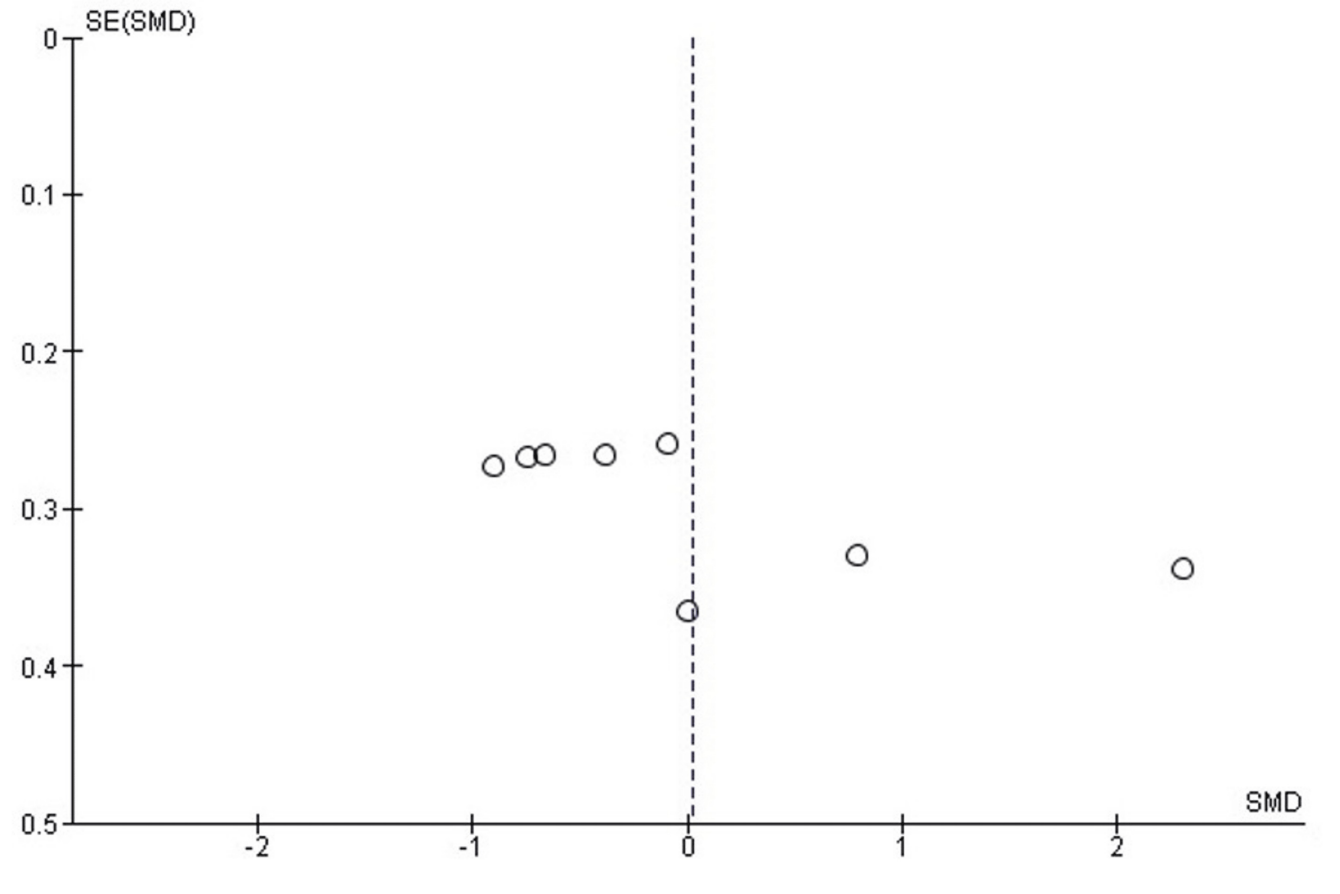
Funnel plot assessing publication bias for pain reduction (VAS) in studies comparing PRP and corticosteroid injections PRP: platelet-rich plasma; VAS: visual analog scale

The funnel plot for pain reduction (VAS score) shows some asymmetry, raising concerns about potential publication bias or heterogeneity among the included studies. However, Egger’s test did not detect significant publication bias, with an intercept of -2.56 and a p-value of 0.512 (p > 0.05). This suggests that the observed asymmetry may be attributed to heterogeneity rather than systematic bias (Figure 4).

Comparison of PRP and Corticosteroid Injections for Function and Pain (ASES Score)

The forest plot evaluates both function and pain levels, measured using the American Shoulder and Elbow Surgeons Standardized Shoulder Assessment Form (ASES), at baseline, 6 weeks, 12 weeks, and 6 months following PRP and CS treatments. At baseline, there was no significant difference between the PRP and CS groups (SMD = 0.02, 95% CI: -0.39 to 0.43, p = 0.92), with moderate heterogeneity (I² = 69%). Similarly, at six weeks, function and pain levels showed no significant differences between groups (SMD = -0.01, 95% CI: -0.30 to 0.27, p = 0.93, I² = 0%).

At 12 weeks, PRP demonstrated a trend toward greater improvement in function and pain levels compared to CS, with an SMD of 0.66 (95% CI: -0.38 to 1.69, p = 0.21), although heterogeneity was high (I² = 88%). At 6 months, PRP showed a more pronounced improvement in ASES scores, with an SMD of 0.35 (95% CI: 0.00 to 0.70, p = 0.05), suggesting a trend toward better long-term outcomes with PRP.

**Figure 5:**
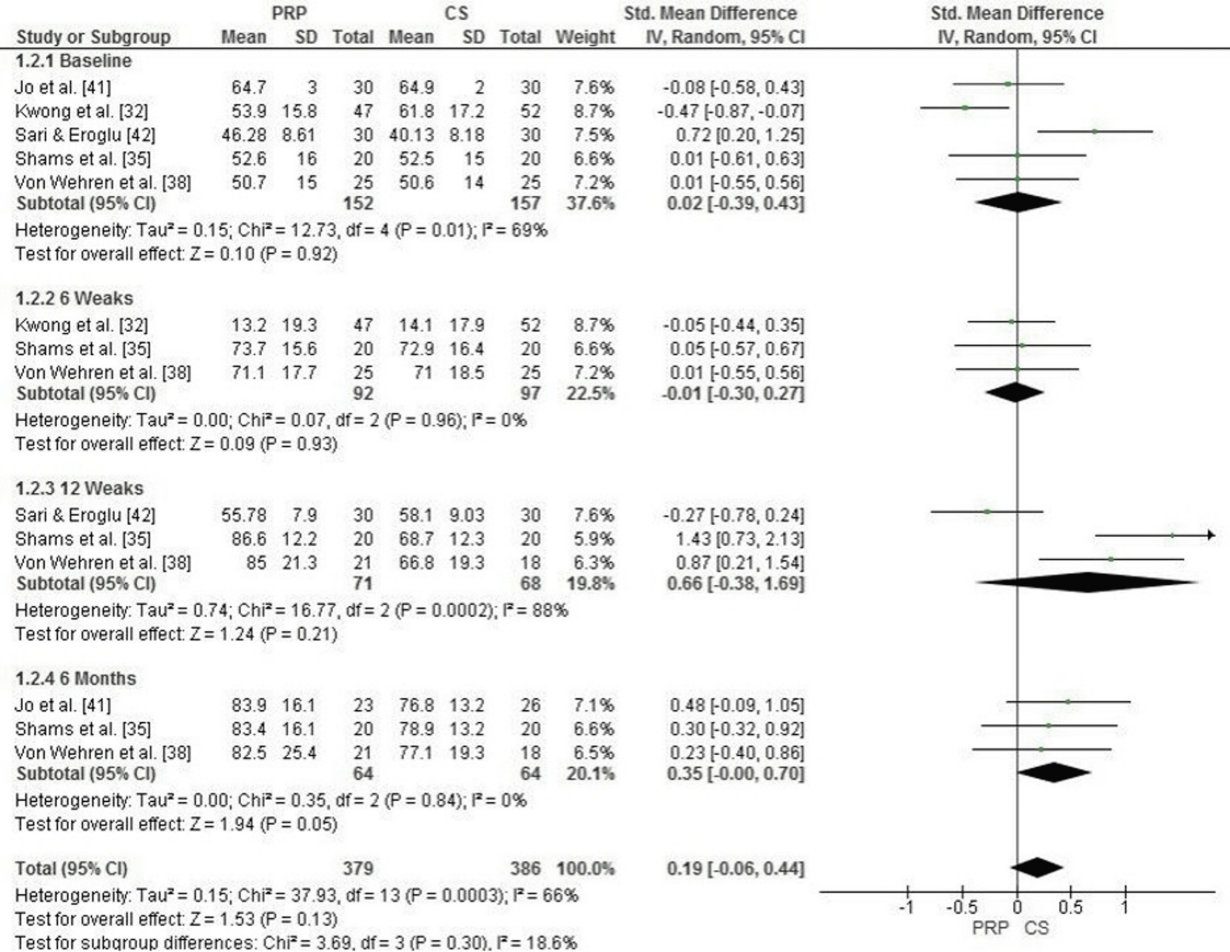
Forest plot comparing PRP and corticosteroid injections for pain and function (ASES score) at baseline, 6 weeks, 12 weeks, and 6 months PRP: platelet-rich plasma; ASES: American Shoulder and Elbow Surgeons score Source: [32,35,38,41,42]

The overall pooled analysis across all time points showed a slight, non-significant favoring of PRP (SMD = 0.19, 95% CI: -0.06 to 0.44, p = 0.13), with moderate heterogeneity (I² = 66%) (Figure 5).

Publication Bias Assessment for Function and Pain (ASES Score)

**Figure 6:**
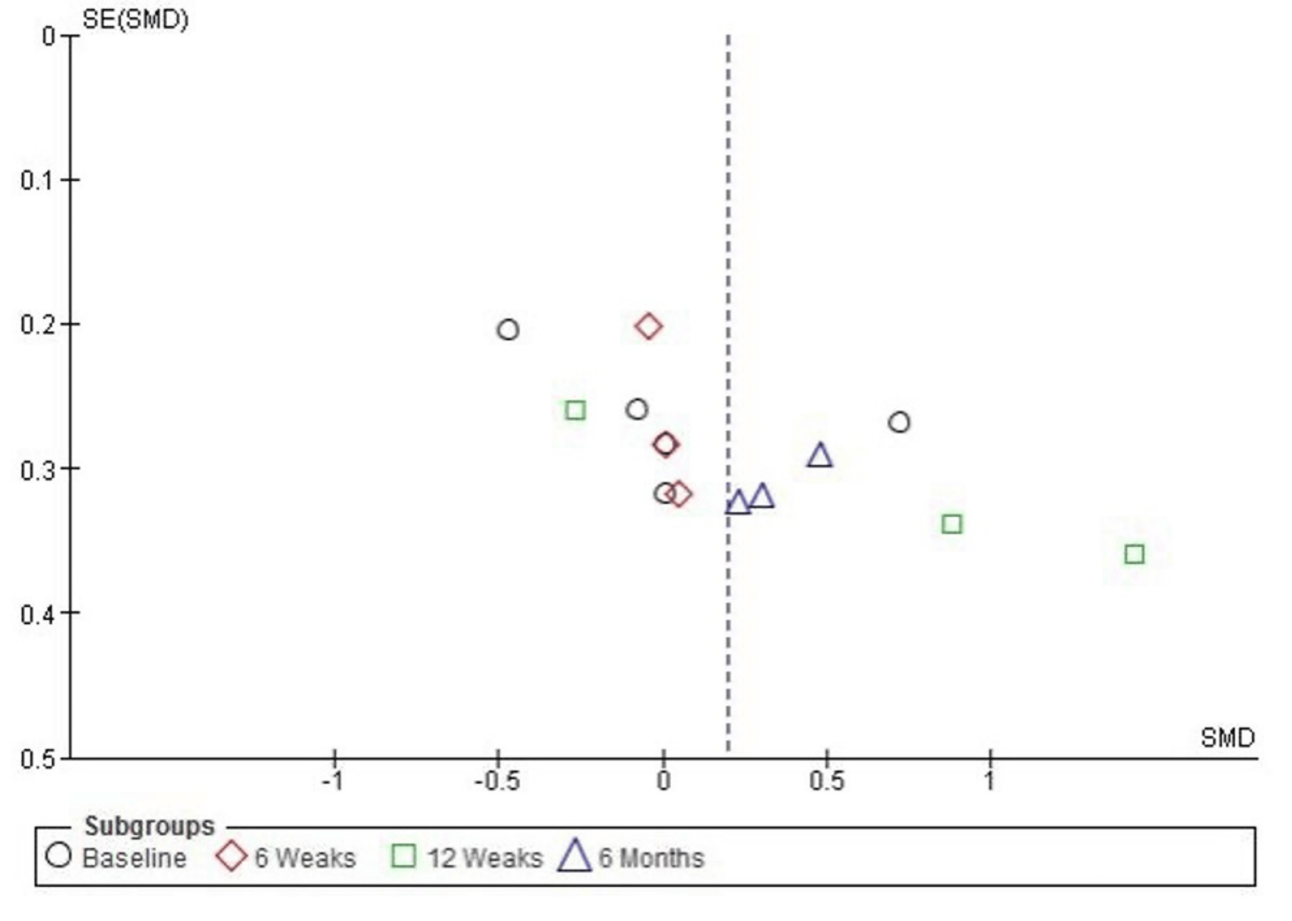
Funnel plot evaluating publication bias for pain and function (ASES score) in studies comparing PRP and corticosteroid injections PRP: platelet-rich plasma; ASES: American Shoulder and Elbow Surgeons score

The funnel plot for function and pain levels, measured by the American Shoulder and Elbow Surgeons (ASES) Standardized Shoulder Assessment Form, indicates some asymmetry, suggesting potential heterogeneity or publication bias. However, Egger’s test did not reveal statistically significant publication bias, with an intercept of 0.67 and a p-value of 0.063 (p > 0.05). This suggests that the observed asymmetry may be attributed to heterogeneity or variability in study methodologies rather than systematic publication bias (Figure 6).

Comparison of PRP and Corticosteroid Injections for Function Improvement (Constant-Murley Score)

The Forest plot evaluates function, measured by the Constant-Murley Score (CMS), across five time points: baseline, 6 weeks, 12 weeks/3 months, 6 months, and 24 months. At baseline, no significant difference was observed between the PRP and CS groups (SMD = 0.06, 95% CI: -0.17 to 0.29, p = 0.59), with low heterogeneity (I² = 13%). At six weeks, CS appeared to perform better, with a pooled SMD of -0.57 (95% CI: -1.31 to 0.17, p = 0.13), although heterogeneity was high (I² = 86%).

**Figure 7:**
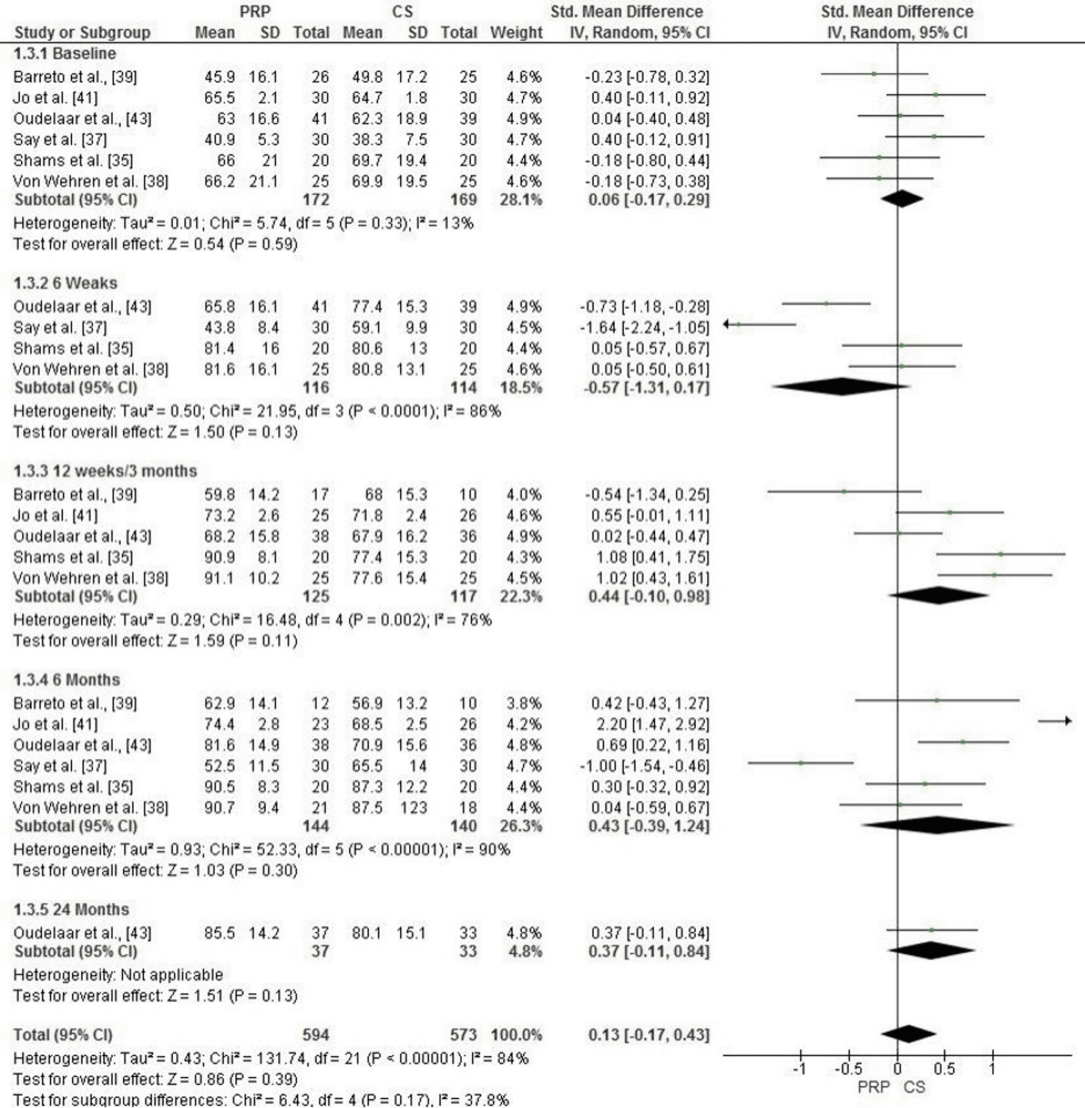
Forest plot comparing PRP and corticosteroid injections for function improvement (Constant-Murley Score) across baseline, 6 weeks, 12 weeks, and 24 months PRP: platelet-rich plasma Source: [35,37-39,41,43]

At 12 weeks/3 months, PRP showed an advantage, with a pooled SMD of 0.44 (95% CI: -0.10 to 0.98, p = 0.11), accompanied by substantial heterogeneity (I² = 76%). At 6 months, PRP maintained its trend toward superior outcomes with an SMD of 0.43 (95% CI: -0.39 to 1.24, p = 0.30), though heterogeneity was very high (I² = 90%). At 24 months, PRP exhibited a slight but non-significant improvement (SMD = 0.37, 95% CI: -0.11 to 0.84, p = 0.13). The overall pooled analysis across all time points favored PRP (SMD = 0.13, 95% CI: -0.17 to 0.43, p = 0.39), but the result was not statistically significant, with substantial overall heterogeneity (I² = 84%) (Figure 7).

Publication Bias Assessment for Function Improvement (Constant-Murley Score)

**Figure 8:**
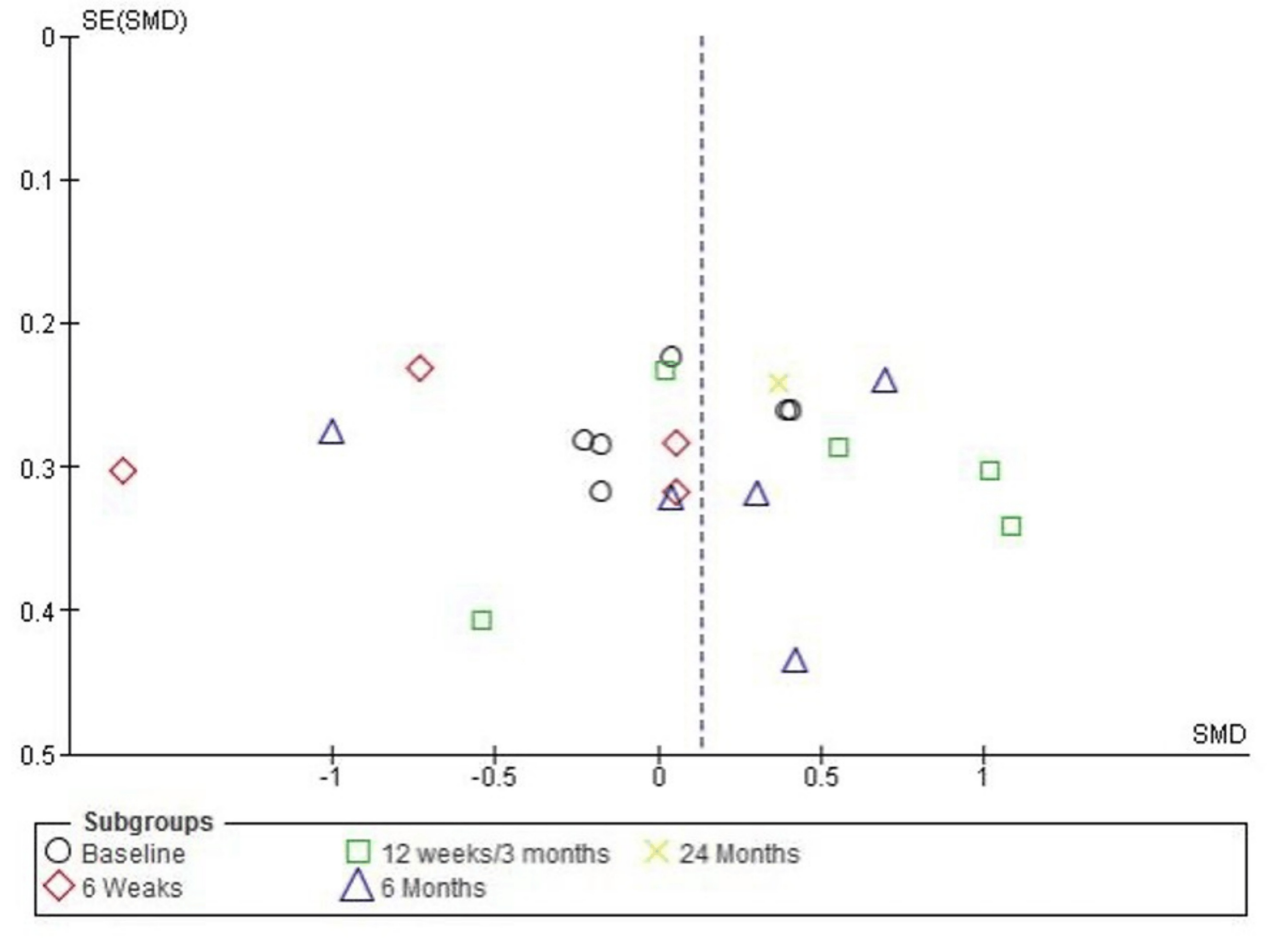
Funnel plot assessing publication bias for function improvement (Constant-Murley Score) in studies comparing PRP and corticosteroid injections PRP: platelet-rich plasma

The funnel plot for function, measured by the Constant-Murley Score (CMS), indicates some asymmetry, suggesting potential heterogeneity among the included studies. However, Egger’s test did not detect statistically significant publication bias, with an intercept of 0.20 and a p-value of 0.464 (p > 0.05). These findings suggest that the observed asymmetry may be attributed to variations in study methodologies, sample sizes, or follow-up durations rather than systematic publication bias. Further high-quality studies are needed to confirm these results (Figure 8).

Comparison of PRP and Corticosteroid Injections for Functional Outcomes (DASH Score)

The Forest plot evaluates function using the DASH score across five time points: baseline, 1 month, 3 months, 6 months, and 12 months. At baseline, no significant difference was observed between PRP and corticosteroid (CS) groups (SMD = 0.20, 95% CI: -0.21 to 0.61, p = 0.33), with moderate heterogeneity (I² = 67%). At 1 month, PRP showed a trend toward better outcomes, with an SMD of 0.40 (95% CI: -0.12 to 0.91, p = 0.13), though heterogeneity was moderate (I² = 61%).

**Figure 9:**
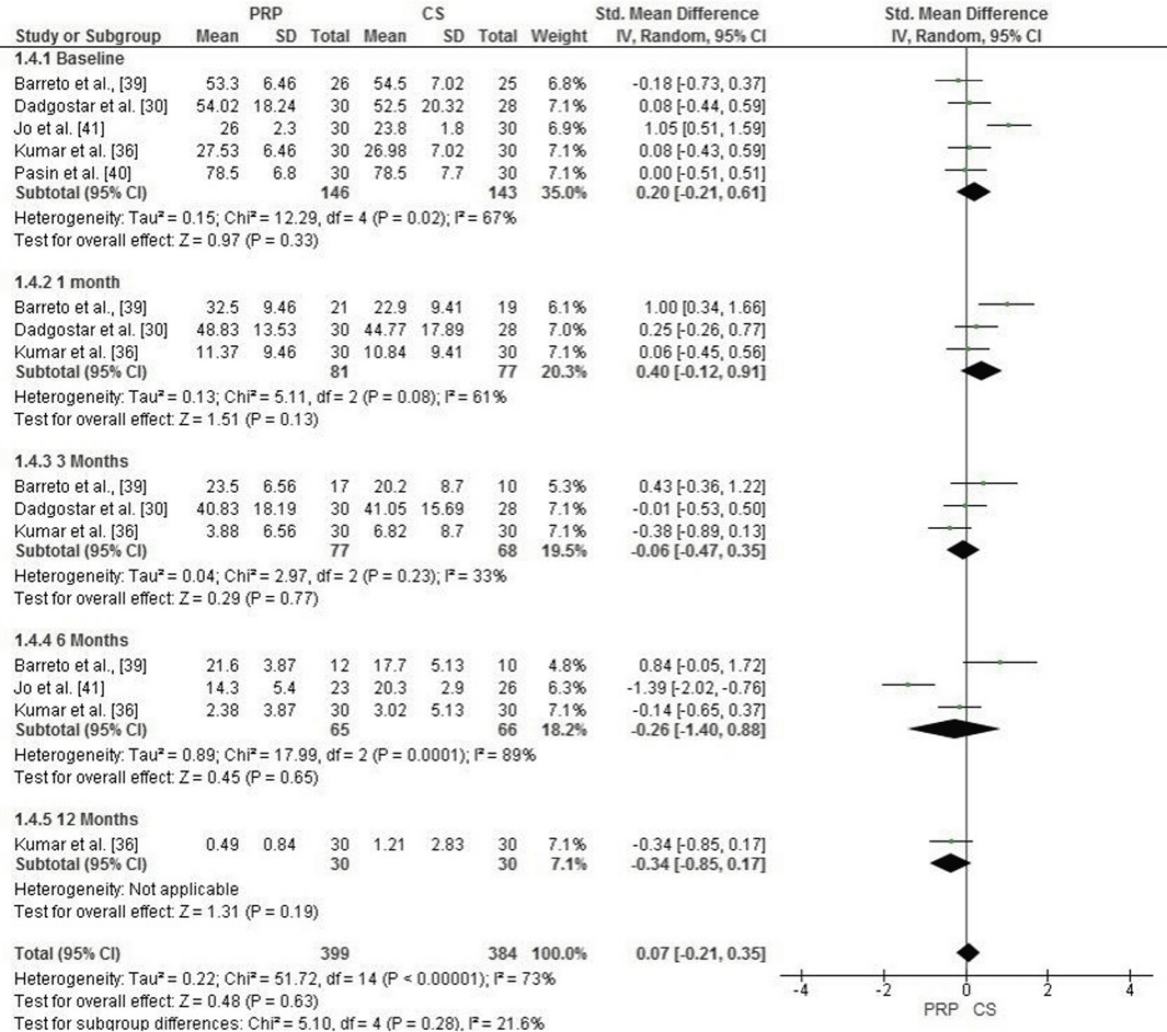
Forest plot comparing PRP and corticosteroid injections for function improvement (DASH score) at baseline, 1 month, 3 months, 6 months, and 12 months. PRP: platelet-rich plasma; DASH: Disabilities of the Arm, Shoulder, and Hand Sources: [30,36,39,40,41]

At 3 months, there was no significant difference between the two groups (SMD = -0.06, 95% CI: -0.47 to 0.35, p = 0.77, I² = 33%). Similarly, at 6 months, PRP showed no advantage, with an SMD of -0.26 (95% CI: -1.40 to 0.88, p = 0.65), and heterogeneity was very high (I² = 89%). At 12 months, the analysis included only one study, which also showed no significant difference (SMD = -0.34, 95% CI: -0.85 to 0.17, p = 0.19). The overall pooled analysis across all time points showed a slight, non-significant benefit for PRP (SMD = 0.07, 95% CI: -0.21 to 0.35, p = 0.60), with substantial heterogeneity (I² = 73%) (Figure 9).

Publication Bias Assessment for Functional Outcomes (DASH Score)

**Figure 10:**
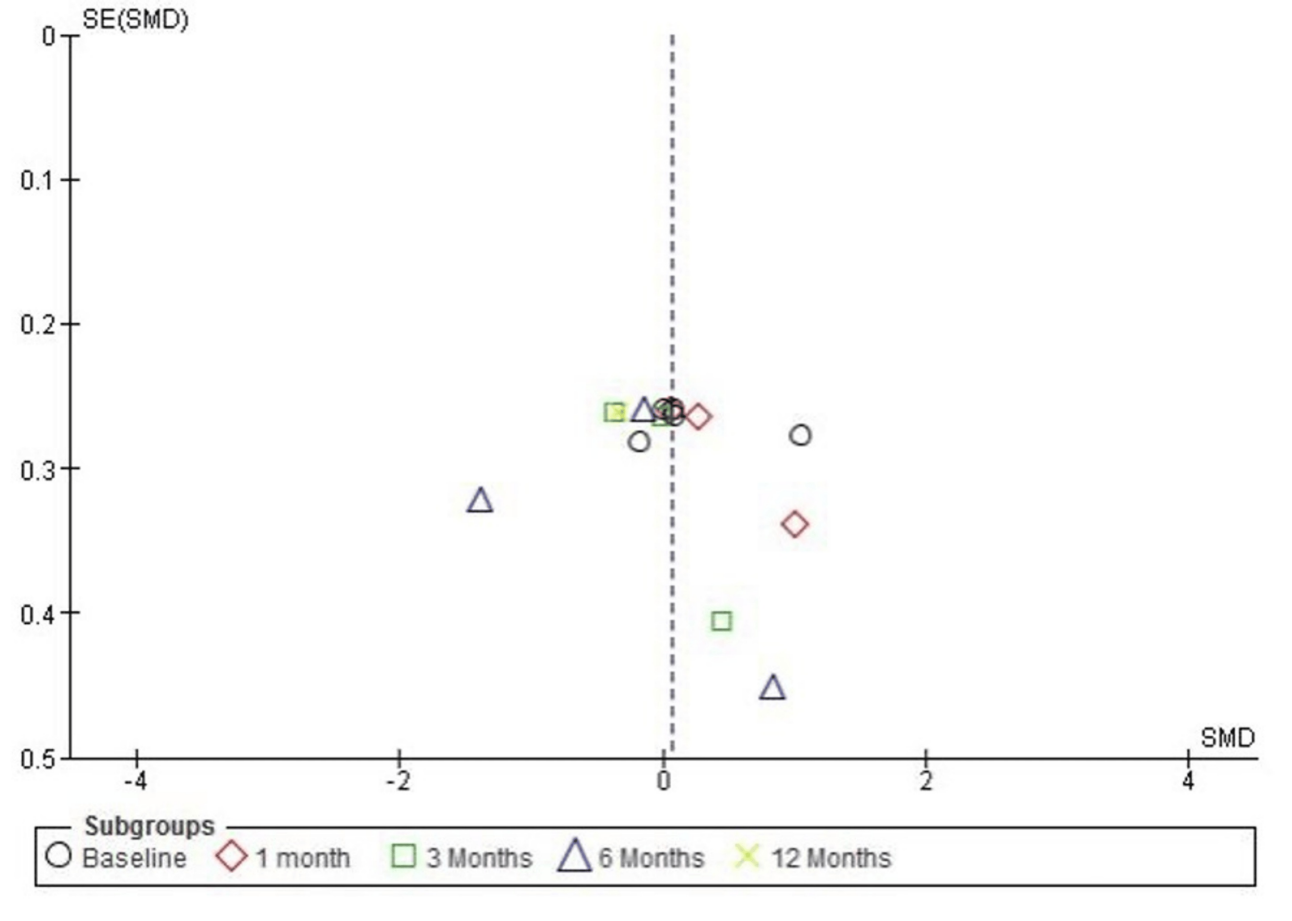
Funnel plot evaluating publication bias for function improvement (DASH score) in studies comparing PRP and corticosteroid injections PRP: platelet-rich plasma; DASH: Disabilities of the Arm, Shoulder, and Hand

The funnel plot for function, measured using the DASH score, suggests slight asymmetry, particularly at longer follow-up durations. However, Egger’s test did not detect a significant publication bias, with an intercept of 0.13 and a p-value of 0.878 (p > 0.05). These results indicate that the observed asymmetry is more likely due to heterogeneity in study methodologies, sample sizes, or follow-up durations rather than a systematic publication bias (Figure 10).

Discussion

The evaluation of platelet-rich plasma (PRP) and corticosteroid (CS) injections in chronic tendinopathies highlights their respective strengths and boundaries. While PRP is rising as a promising long-term healing option, CS remains a famous preference for immediate pain comfort. This meta-analysis provides a nuanced expertise of their efficacy, presenting insights into trends and aligning or diverging findings from earlier studies.

Our meta-analysis showed no significant difference between PRP and CS in pain reduction (SMD = 0.12, 95% CI: -0.64 to 0.88, p = 0.75), with vast heterogeneity (I² = 92%). These results align with studies with the aid of Miller et al., who found mild efficacy for PRP but emphasized its variability [45]. Lin et al. also stated comparable brief-term pain comfort for PRP and CS in rotator cuff tendinopathy, consistent with our findings [46]. However, Say et al. [37] and Sabaah & Nassif [44] suggest that PRP may provide effective pain relief in some populations, especially those with long-term follow-up. Fitzpatrick et al. reported a significant improvement in pain scores in PRP at 24 weeks, which outweighed the effect of CS, which declined over time [47].

Our findings show no significant difference between PRP and CS in terms of improved function and pain as measured by ASES scores at baseline and short-term follow-up such as at 6 weeks (SMD = -0.01, 95% CI: -0.30 to 0.27, p = 0.93). These results are consistent with those obtained by a study by Kwong et al. that reported no significant difference between PRP and CS in the early postinjection period [48]. PRP at 12 weeks showed a trend toward optimal results (SMD = 0.66, 95% CI: -0.38 to 1.69, p = 0.21), and the improvement at 6 months was marginally significant (SMD = 0.35, 95% CI: 0.00 to 0.70, p = 0.05). This delay in PRP is consistent with the findings of Fitzpatrick et al. who reported a consistent effect of PRP improving function at long-term follow-up [49]. In contrast, CS tends to show rapid but transient benefits, consistent with the findings of Dadgostar et al. [30].

In functional outcomes measured by the Constant-Murley score (CMS), no significant difference was found between PRP and CS at all time points (SMD = 0.13, 95% CI: -0.17 to 0.43, p = 0.39). However, PRP showed a slight benefit at 24 months (SMD = 0.37, 95% CI: -0.11 to 0.84, p = 0.13), although the effect was not statistically significant. Ibrahim et al. also reported that PRP provides better long-term functional recovery compared to CS [33]. In some studies, the transient elevation of CS can be attributed to anti-inflammatory effects, resulting in immediate relief of symptoms. However, these effects are usually inhibited by muscle weakness, according to Saleem et al. [34], who emphasized the increased risk of vascular rupture with repeated CS injections.

Functional outcomes examined by the Disabilities of the Arm, Shoulder, and Hand (DASH) scores showed no significant difference between PRP and CS for most of the time period, with a slight, non-significant trend favoring PRP (SMD = 0.07, 95% CI: -0.21 to 0.35, p = 0.60). Similarly, no significant differences in DASH scores were reported between PRP and CS, especially during the first follow-up period.

The observed differences in funnel plots across the results suggest potential heterogeneity due to study methods, sample size, and follow-up time; however, Egger’s test could not detect significant publication bias consistently at all assessments (e.g., p > 0.05 for VAS, ASES, CMS, and DASH scores). By this, it is known that heterogeneity may be due to differences in study design rather than systematic bias.

Our findings suggest that although PRP and CS provide comparable short-term pain relief, PRP may provide limited long-term benefits in terms of function and pain reduction. However, variability in PRP manufacturing and application techniques limits its overall clinical application. Future research should focus on standardizing the PRP protocol and conducting larger randomized controlled trials to provide more conclusive evidence of its comparative efficacy.

Limitations

Several limitations of the current literature must be considered. Many of the studies included in this review had small sample sizes, were unblinded, or had limited short follow-up periods. In addition, significant heterogeneity in PRP preparation techniques (e.g., leukocyte-rich vs. leukocyte-poor) and corticosteroid formulations makes comparisons between studies difficult. The absence of standardized protocols further limits the ability to draw definitive conclusions about the superiority of one intervention over the other.

## Conclusions

This systematic review and meta-analysis examined the efficacy of platelet-rich plasma (PRP) and corticosteroid (CS) injections for chronic tendinopathies, focusing on pain control and functional improvement in a variety of validated metrics. Although individual studies sometimes demonstrated distinct advantages of PRP over CS, the overall findings showed comparable results for most parameters. However, PRP showed a slight tendency to provide better long-term improvement in functional outcomes. Despite this, the evidence remains inconclusive due to significant differences in reporting of PRP protocols and outcomes. Future high-quality, large-scale randomized trials with standardized PRP regimens, longer follow-up periods, and comprehensive clinical guidelines are needed to clarify its function.

## References

[1] Lui PP (2017). Tendinopathy in diabetes mellitus patients-epidemiology, pathogenesis, and management. Scand J Med Sci Sports.

[2] Riel H, Lindstrøm CF, Rathleff MS, Jensen MB, Olesen JL (2019). Prevalence and incidence rate of lower-extremity tendinopathies in a Danish general practice: a registry-based study. BMC Musculoskelet Disord.

[3] Cannata F, Vadalà G, Ambrosio L, Napoli N, Papalia R, Denaro V, Pozzilli P (2021). The impact of type 2 diabetes on the development of tendinopathy. Diabetes Metab Res Rev.

[4] Kwan CK, Fu SC, Yung PS (2020). A high glucose level stimulate inflammation and weaken pro-resolving response in tendon cells - a possible factor contributing to tendinopathy in diabetic patients. Asia Pac J Sports Med Arthrosc Rehabil Technol.

[5] Malliaras P, O’Neill S (2017). Potential risk factors leading to tendinopathy. Apunts Med Esport.

[6] Vasta S, Di Martino A, Zampogna B, Torre G, Papalia R, Denaro V (2016). Role of VEGF, nitric oxide, and sympathetic neurotransmitters in the pathogenesis of tendinopathy: a review of the current evidences. Front Aging Neurosci.

[7] Chisari E, Rehak L, Khan WS, Maffulli N (2019). Tendon healing in presence of chronic low-level inflammation: a systematic review. Br Med Bull.

[8] Tran PH, Malmgaard-Clausen NM, Puggaard RS (2020). Early development of tendinopathy in humans: sequence of pathological changes in structure and tissue turnover signaling. FASEB J.

[9] Fu SC, Yeung MY, Rolf CG, Yung PS, Chan KM, Hung LK (2018). Hydrogen peroxide induced tendinopathic changes in a rat model of patellar tendon injury. J Orthop Res.

[10] Irby A, Gutierrez J, Chamberlin C, Thomas SJ, Rosen AB (2020). Clinical management of tendinopathy: a systematic review of systematic reviews evaluating the effectiveness of tendinopathy treatments. Scand J Med Sci Sports.

[11] Gerdesmeyer L, Mittermayr R, Fuerst M, Al Muderis M, Thiele R, Saxena A, Gollwitzer H (2015). Current evidence of extracorporeal shock wave therapy in chronic Achilles tendinopathy. Int J Surg.

[12] Yang SM, Chen WS (2020). Conservative treatment of tendon injuries. Am J Phys Med Rehabil.

[13] Stoychev V, Finestone AS, Kalichman L (2020). Dry needling as a treatment modality for tendinopathy: a narrative review. Curr Rev Musculoskelet Med.

[14] Aicale R, Bisaccia RD, Oliviero A, Oliva F, Maffulli N (2020). Current pharmacological approaches to the treatment of tendinopathy. Expert Opin Pharmacother.

[15] Akoh CC, Phisitkul P (2019). Minimally invasive and endoscopic approach for the treatment of noninsertional Achilles tendinopathy. Foot Ankle Clin.

[16] Everhart JS, Cole D, Sojka JH, Higgins JD, Magnussen RA, Schmitt LC, Flanigan DC (2017). Treatment options for patellar tendinopathy: a systematic review. Arthroscopy.

[17] Wu PI, Diaz R, Borg-Stein J (2016). Platelet-rich plasma. Phys Med Rehabil Clin N Am.

[18] Shahid M, Kundra R (2017). Platelet-rich plasma (PRP) for knee disorders. EFORT Open Rev.

[19] Marques LF, Stessuk T, Camargo IC, Sabeh Junior N, dos Santos L, Ribeiro-Paes JT (2015). Platelet-rich plasma (PRP): methodological aspects and clinical applications. Platelets.

[20] Everts P, Onishi K, Jayaram P, Lana JF, Mautner K (2020). Platelet-rich plasma: new performance understandings and therapeutic considerations in 2020. Int J Mol Sci.

[21] Gupta S, Paliczak A, Delgado D (2021). Evidence-based indications of platelet-rich plasma therapy. Expert Rev Hematol.

[22] Chen NF, Sung CS, Wen ZH (2018). Therapeutic effect of platelet-rich plasma in rat spinal cord injuries. Front Neurosci.

[23] Ehrenfest D, Andia I, Zumstein M (2014). Classification of platelet concentrates (platelet-rich plasma-PRP, platelet-rich fibrin-PRF) for topical and infiltrative use in orthopedic and sports medicine: current consensus, clinical implications, and perspectives. Muscles Ligaments Tendons.

[24] Alsousou J, Harrison P (10.1016/B978-0-12-813456-6.00065-5). Therapeutic platelet-rich plasma in wound healing. Platelets (Fourth Edition).

[25] Williams DM (2018). Clinical pharmacology of corticosteroids. Respir Care.

[26] Ferrara G, Petrillo MG, Giani T (2019). Clinical use and molecular action of corticosteroids in the pediatric age. Int J Mol Sci.

[27] Al-Mosawi R, Alebadi N (2023). Usage of corticosteroids as therapeutic agents in diseases. Med J Babylon.

[28] Stone S, Malanga GA, Capella T (2021). Corticosteroids: review of the history, the effectiveness, and adverse effects in the treatment of joint pain. Pain Physician.

[29] Mousavizadeh R, Backman L, McCormack RG, Scott A (2015). Dexamethasone decreases substance P expression in human tendon cells: an in vitro study. Rheumatology (Oxford).

[30] Dadgostar H, Fahimipour F, Pahlevan Sabagh A, Arasteh P, Razi M (2021). Corticosteroids or platelet-rich plasma injections for rotator cuff tendinopathy: a randomized clinical trial study. J Orthop Surg Res.

[31] Wernecke C, Braun HJ, Dragoo JL (2015). The effect of intra-articular corticosteroids on articular cartilage: a systematic review. Orthop J Sports Med.

[32] (2024). The Cochrane Collaboration. Review Manager (RevMan) Version 5.4. https://training.cochrane.org/system/files/uploads/protected_file/RevMan5.4_user_guide.pdf.

[33] Ibrahim DH, El-Gazzar NM, El-Saadany HM (2019). Ultrasound-guided injection of platelet-rich plasma versus corticosteroid for treatment of rotator cuff tendinopathy: effect on shoulder pain, disability, range of motion and ultrasonographic findings. Egyptian Rheumatol.

[34] Saleem U, Afzal MK, Saqib M, Azam MF (2022). Comparison of local corticosteroids versus plasma rich protein for management of rotator cuff tendinopathy. Pak J Med Health Sci.

[35] Shams A, El-Sayed M, Gamal O, Ewes W (2016). Subacromial injection of autologous platelet-rich plasma versus corticosteroid for the treatment of symptomatic partial rotator cuff tears. Eur J Orthop Surg Traumatol.

[36] Kumar V, Talwar J, Rustagi A, Krishna LG, Sharma VK (2023). Comparison of clinical and functional outcomes after platelet-rich plasma injection and corticosteroid injection for the treatment of de Quervain's tenosynovitis. J Wrist Surg.

[37] Say F, Gurler D, Bulbul M (2016). Platelet-rich plasma versus steroid injection for subacromial impingement syndrome. J Orthop Surg (Hong Kong).

[38] von Wehren L, Blanke F, Todorov A, Heisterbach P, Sailer J, Majewski M (2016). The effect of subacromial injections of autologous conditioned plasma versus cortisone for the treatment of symptomatic partial rotator cuff tears. Knee Surg Sports Traumatol Arthrosc.

[39] Barreto RB, Azevedo AR, Gois MC, Freire MR, Silva DS, Cardoso JC (2019). Platelet-rich plasma and corticosteroid in the treatment of rotator cuff impingement syndrome: randomized clinical trial. Rev Bras Ortop (Sao Paulo).

[40] Pasin T, Ataoğlu S, Pasin Ö, Ankarali H (2019). Comparison of the effectiveness of platelet-rich plasma, corticosteroid, and physical therapy in subacromial impingement syndrome. Arch Rheumatol.

[41] Jo CH, Lee SY, Yoon KS, Oh S, Shin S (2020). Allogeneic platelet-rich plasma versus corticosteroid injection for the treatment of rotator cuff disease: a randomized controlled trial. J Bone Joint Surg Am.

[42] Sari A, Eroglu A (2020). Comparison of ultrasound-guided platelet-rich plasma, prolotherapy, and corticosteroid injections in rotator cuff lesions. J Back Musculoskelet Rehabil.

[43] Oudelaar BW, Huis In 't Veld R, Ooms EM, Schepers-Bok R, Nelissen RG, Vochteloo AJ (2021). Efficacy of adjuvant application of platelet-rich plasma after needle aspiration of calcific deposits for the treatment of rotator cuff calcific tendinitis: a double-blinded, randomized controlled trial with 2-year follow-up. Am J Sports Med.

[44] Sabaah HMAE, Nassif MA (2020). What is better for rotator cuff tendinopathy: dextrose prolotherapy, platelet-rich plasma, or corticosteroid injections? A randomized controlled study. Egyptian Rheumatol Rehabil.

[45] Miller LE, Parrish WR, Roides B, Bhattacharyya S (2017). Efficacy of platelet-rich plasma injections for symptomatic tendinopathy: systematic review and meta-analysis of randomised injection-controlled trials. BMJ Open Sport Exerc Med.

[46] Lin MT, Chiang CF, Wu CH, Huang YT, Tu YK, Wang TG (2019). Comparative effectiveness of injection therapies in rotator cuff tendinopathy: a systematic review, pairwise and network meta-analysis of randomized controlled trials. Arch Phys Med Rehabil.

[47] Fitzpatrick J, Bulsara MK, O'Donnell J, Zheng MH (2019). Leucocyte-rich platelet-rich plasma treatment of gluteus medius and minimus tendinopathy: a double-blind randomized controlled trial with 2-year follow-up. Am J Sports Med.

[48] Fitzpatrick J, Bulsara MK, O'Donnell J, McCrory PR, Zheng MH (2018). The effectiveness of platelet-rich plasma injections in gluteal tendinopathy: a randomized, double-blind controlled trial comparing a single platelet-rich plasma injection with a single corticosteroid injection. Am J Sports Med.

[49] Kwong CA, Woodmass JM, Gusnowski EM, Bois AJ, Leblanc J, More KD, Lo IK (2021). Platelet-rich plasma in patients with partial-thickness rotator cuff tears or tendinopathy leads to significantly improved short-term pain relief and function compared with corticosteroid injection: a double-blind randomized controlled trial. Arthroscopy.

